# Higher Education Instructors’ Usage of and Learning From Student Evaluations of Teaching – Do Achievement Goals Matter?

**DOI:** 10.3389/fpsyg.2021.652093

**Published:** 2021-07-20

**Authors:** Julia Hein, Stefan Janke, Raven Rinas, Martin Daumiller, Markus Dresel, Oliver Dickhäuser

**Affiliations:** ^1^Department of Social Sciences, University of Mannheim, Mannheim, Germany; ^2^Department of Psychology, University of Augsburg, Augsburg, Germany

**Keywords:** achievement goals, instructors, professional learning, student evaluations of teaching, higher education

## Abstract

Identifying what motivates and hinders higher education instructors in their self-regulated learning from student evaluations of teaching (SETs) is important for improving future teaching and facilitating student learning. According to models of self-regulated learning, we propose a model for the usage of SETs as a learning situation. In a longitudinal study, we investigate the associations between achievement goals and the usage of and learning from SETs in the context of higher education. In total, 407 higher education instructors (46.4% female; 38.60 years on average) with teaching commitments in Germany or Austria reported their achievement goals in an online survey. Out of these participants, 152 instructors voluntarily conducted SET(s) and subsequently reported their intentions to act on the feedback and improve future teaching in a short survey. Using structural equation modeling, we found, in line with our hypotheses, that learning avoidance, appearance approach, and appearance avoidance goals predicted whether instructors voluntarily conducted SET(s). As expected, learning approach and (avoidance) goals were positively associated with intentions to act on received SET-results and improve future teaching. These findings support our hypotheses, are in line with assumptions of self-regulated learning models, and highlight the importance of achievement goals for instructors’ voluntary usage of and intended learning from SET(s). To facilitate instructors’ learning from SET-results, our study constitutes a first step for future intervention studies to build on. Future researchers and practitioners might support instructors’ professional learning by encouraging them to reflect on their SET-results.

## Introduction

Student evaluations of teaching (SETs) are used in a wide range of universities and higher education institutions as a tool to provide valuable feedback to instructors (Marsh and Roche, 1993; Wagenaar, 1995; Zhao and Gallant, 2012) and serve the purpose of improving teaching quality (Nowakowski and Hannover, 2015). The implementation of SETs can improve teaching effectiveness (Serin, 2019), especially if the feedback given in the SETs is complemented by external consultation (Marsh, 1984; Marsh and Roche, 1993). Nevertheless, the impact of SETs likely depends on instructors’ willingness to use and process student feedback for the development of their teaching (Kember et al., 2002). From our view, instructors should have a proactive role in generating and using feedback, similar to assumptions regarding students (Molloy and Boud, 2013; see Boud and Molloy, 2013). Therefore, we raise the question of what individual characteristics might prevent instructors from using SETs for the improvement of their teaching. To this end, little research has been conducted thus far concerning how instructors process SET-results (Nowakowski and Hannover, 2015) or the factors that trigger their intentions to learn from and act on SET-results and improve their teaching behavior. Such research is important as instructors need to actively engage with feedback in the form of SETs by interpreting and internalizing the given information to develop their teaching (as discussed for the use of external feedback to enhance performance in school students, see Ivanic et al., 2000; Nicol and Macfarlane-Dick, 2006). This active engagement in SETs that represents a self-regulated learning process mandates motivation, which resonates well with the emerging evidence that university instructors’ achievement goals for teaching are associated with their engagement in professional learning (Daumiller et al., 2021c). Particularly, learning goals (i.e., striving to develop professional competencies) predict professional learning (Diethert et al., 2015; Hein et al., 2019, 2020, see also Nitsche et al., 2013 for school teachers). Here, we propose a model that might explain why and how university instructors’ teaching-related achievement goals are important predictors for the use of SETs, processing of SET-results, and intentions to improve teaching.

### Achievement Goals as Antecedents of Learning With and From Sets

*Achievement goals* are future-focused cognitive representations of competence-related results or end states that an individual is committed to either approach or avoid (Payne et al., 2007; Hulleman et al., 2010). In line with prior research in the teaching domain, we distinguish between learning approach (focus on developing competence), learning avoidance (focus on avoiding not developing own competencies to the fullest extent), performance approach (focus on being perceived as competent), performance avoidance (focus on avoiding appearing incompetent), and work avoidance (focus on effort reduction by engaging in tasks with as little effort as possible) goals (see Butler and Shibaz, 2008; Retelsdorf et al., 2010; Butler, 2014; Daumiller et al., 2019). Although research investigating higher education instructors’ achievement goals is still a young field of research (Daumiller et al., 2020), there is first evidence that higher education instructors’ achievement goals guide their behavior (e.g., teaching quality and professional development) and predict emotions as well as cognitions (Diethert et al., 2015; Janke and Dickhäuser, 2018; Daumiller et al., 2019; Hein et al., 2019; Rinas et al., 2020).

Regarding the usage and processing of SETs, achievement goals may act as a lens that filters the perception of students’ feedback as a potential asset or obstacle for goal striving (in line with Nicol and Macfarlane-Dick, 2006; Kaplan and Maehr, 2007). Consequently, achievement goals might explain how instructors interpret the feedback situation (e.g., as a learning opportunity, an opportunity to appear competent, a risk of appearing incompetent, or an effort that could be reduced) and how they profit from student feedback. This impact of achievement goals could occur in different phases of the self-regulated learning process. Even if SETs are typically mandatory at higher education institutions, instructors still need to process the SETs on their own and use the results to evaluate potential effects of their goal striving.

Models of self-regulated learning differentiate between pre-action (forethought), action (performance), and post-action (reflection) phases of the learning process (e.g., Zimmerman, 2000; Schmitz and Wiese, 2006). In our study, we focus on voluntarily conducted SETs to include all phases of the learning process. In the pre-action phase, instructors’ motivation determines the initiation of the learning activity by deciding and planning to conduct voluntary SETs. Here, it seems particularly important whether or not instructors see SETs as beneficial tools for their goal striving. During the action phase, instructors process the SET-results and likely need to interpret how these results align with their own achievement goals to draw relevant conclusions for their teaching (Butler and Winne, 1995; Nicol and Macfarlane-Dick, 2006). Finally, in the post-action phase, instructors reflect on what they have learned and form intentions about how to further improve their teaching in a way that helps them to reach their achievement goals. These intentions concerning the SET-results may eventually lead to changes in actual teaching behavior, and in turn, teaching quality (in line with the theory of planned behavior, Ajzen, 1991; Achtziger and Gollwitzer, 2018). Prior research supports this association between intentions and behaviors (Webb and Sheeran, 2006; Hurtz and Williams, 2009). In the following section, we will discuss how achievement goals impact the different phases of self-regulated learning, as the learning result is dependent upon on instructors’ engagement (and motivation) in each of these phases (Zimmerman, 2000; Schmitz and Wiese, 2006).

### Different Types of Achievement Goals and Learning From SETs

Learning approach goals facilitate the active search for learning opportunities, which is critical for the development of competencies. Indeed, prior studies have shown that learning approach goals are closely tied to actual and intended engagement regarding formal and informal learning behaviors in a variety of contexts (Choi and Jacobs, 2011; Nitsche et al., 2013; Diethert et al., 2015; Cerasoli et al., 2018). More specifically, learning approach goals (a component of mastery goals) are positively associated with the intention to participate in formal trainings of employees in academia (see Diethert et al., 2015; Fritzsche and Daumiller, 2018), and teachers’ intentions to implement new curriculum (Gorozidis and Papaioannou, 2011) in the pre-action phase. In addition, learning approach goals are related to engagement within formal professional training courses (action phase, see Daumiller et al., 2021c), school teachers’ help-seeking behavior (action phase, see Butler, 2007; Dickhäuser et al., 2007), school teachers’ asking for feedback and reflection (action and post-action phases, Runhaar et al., 2010), as well as with learning results in adult samples (post-action phase, Payne et al., 2007). As such, we assume that learning approach goals will have a beneficial impact on all steps of self-regulated learning. For SETs, this means that we can assume that learning approach goals are associated with instructors’ willingness to conduct SETs (and ask their students for feedback), their effort to process the feedback, their intentions to act on SET-results, as well as their intention to improve future teaching. While learning avoidance goals have sparked scientific debate about their relevance for learning processes (Cury et al., 2006; Hulleman et al., 2010), prior research has suggested that they may be beneficial for instructors’ teaching and professional learning (Daumiller et al., 2019; Hein et al., 2020). We consider it to be a distinct possibility that the striving to avoid missing a learning opportunity could enhance instructors’ vigor to voluntarily conduct SET(s) (in the pre-action phase), process students’ SET-results (in the action phase), and derive further intentions to act on the SET-results and improve future teaching (in the post-action phase).

Asking students for feedback through SETs does not only constitute a learning situation, but also a performance situation for instructors. Specifically, we assume that SETs may help instructors to comprehend whether they appear competent in the eyes of their students (appearance is a core component of instructors’ performance goals, see Daumiller et al., 2019)^1^. Performance approach goals can be seen as a preference to attain favorable judgments of teaching-related competence which is grounded in high competence expectancies, whereas performance avoidance goals might be interpreted as a preference to avoid unfavorable judgments (Elliot and Church, 1997). This means that performance approach goals could motivate instructors to engage in SETs to receive praise, whereas performance avoidance goals could motivate them to abstain from using SETs, given the danger of receiving self-diminishing feedback. Empirical studies support this assumption in samples of school teachers, as performance approach goals have been associated with positive perceptions of help-seeking (Nitsche et al., 2011), and performance avoidance goals have been related to negative perceptions of help-seeking and avoidance of help (Butler, 2007; Dickhäuser et al., 2007; Nitsche et al., 2011). In sum, we consider both performance approach and avoidance goals as predictors for the initiation of the learning process (pre-action phase). However, we do not have directed hypotheses regarding the association of performance goals and the processing of SET-results (action phase). In addition, we do not expect performance goals to facilitate further intentions to act on SET-results or intentions to improve teaching (post-action phase), congruent with prior research on adult learning and teachers’ intentions (Payne et al., 2007; Gorozidis and Papaioannou, 2011).

Finally, a negative association between work avoidance goals and learning from SETs is highly plausible. Since all necessary steps for using SETs and learning from their results can be considered to be effortful in nature, teaching-related work avoidance goals should be detrimental for the whole learning process. In line with this assumption, empirical studies with school teachers suggest that work avoidance goals are associated with a lower number of attended training workshops (Nitsche et al., 2013), the perception of help-seeking as effortful and preference for expedient help seeking (Butler, 2007; Dickhäuser et al., 2007), less engagement, and less self-reported learning gains of higher education instructors in professional training courses (Daumiller et al., 2021c) in the action and post-action phases.

### Mediation Processes in Self-Regulated Learning From SETs

Following models of self-regulated learning (Zimmerman, 1990; Schmitz and Wiese, 2006), we assume that the impact that motivation (here, in form of achievement goals) has on early phases of the learning process also impacts the later phases. In other words, if achievement goals hinder instructors to engage in SETs, they cannot process SETs in the first place. Moreover, if instructors invest more effort to process SETs, they should also find more possibilities to improve future teaching and might be more willing to act on the processed SET-results. While it is trivial that the lack of student feedback in the form of SETs directly corresponds to being unable to process students’ feedback, the association between processing and derived intentions should be further tested. We expect such mediation processes to be important for the impact of learning goals, which are meant to provide the necessary motivation to develop intentions based on the information in the SETs. Moreover, the maladaptive impact of work avoidance goals on intentions to act on SET-results and to improve future teaching might be mediated through an insufficient processing of SET-results. We do not expect such mediation processes for performance goals.

Prior research supports the existence of mediation processes alongside the assumption of models of self-regulated learning. In student samples, positive associations between motivation and performance have been mediated by engagement using video hits as an objective, quantitative measure in massive open online courses (de Barba et al., 2016). Student teachers’ acquisition of pedagogical knowledge (post-action phase) has also been found to depend on the usage of learning opportunities in the action phase (Watson et al., 2018). For instructors specifically, studies have shown that learning engagement (in the form of intensity and elaboration) mediates the associations between learning approach goals/work avoidance goals and learning gains within professional training courses (Daumiller et al., 2021c). Self-reported learning time for formal and informal leaning activities has also been found to mediate the positive associations of learning (approach/avoidance) goals with self-reported learning results (Hein et al., 2019, 2020). Moreover, learning goals have been positively and work avoidance goals negatively related to observed attention (Kücherer et al., 2020). However, the informative value of prior research on this mediation process within samples of instructors may be limited by the same method bias, as most constructs were assessed by self-report-measures. In our research, we thereby want to show that mediation processes which bridge different phases of self-regulated learning exist by using objective indicators of the learning activity to overcome these methodological limitations in research on instructors’ professional learning.

### Moderators of the Impact of Achievement Goals on Learning From SETs

The validity of SETs is strongly debated within the literature and, as such, also among higher education instructors (Marsh, 1984; Spooren and Mortelmans, 2006; Hornstein, 2017). As a result, instructors may differ in their beliefs about SETs to be appropriate measures of teaching quality that can be used as tools to advance their teaching or not. Such beliefs may thereby influence whether instructors voluntarily use SETs. Beliefs can be seen as conditional knowledge that can be interpreted as if-then rules (Butler and Winne, 1995). If instructors believe in the validity of SETs, then they should be more likely to rely on them, as they consider students’ feedback to constitute valid and realistic information about their teaching quality. However, if instructors believe that students cannot assess teaching quality, then they will not ask students for their opinion on their performance in class in the first place. Besides direct effects on SET-usage, we also assume that instructors’ beliefs in the validity of SETs may moderate effects of achievement goals. Specifically, SETs can only be seen as learning opportunities if instructors believe in the validity of student evaluations. Therefore, the positive link between learning goals and the use of SETs should be stronger given these validity beliefs. If, however, SETs are not seen as valid judgments, learning goals should not affect the decision to ask students for feedback.

Additionally, instructors may differ in the degree of psychological threat that they experience from negative feedback. This could have direct, negative effects on the likelihood of using SETs, and at the same time, might also moderate the impact of learning goals. If the general experience of threat through negative feedback is strong, this might hinder instructors from pursuing their learning goals by asking their students for feedback, as this situation entails the possibility of attaining negative judgments. This may especially be the case when considering that instructors could use other learning opportunities to improve their teaching and pursue their learning goals (e.g., formal learning opportunities such as didactical courses). To sum up, the general experienced threat through negative feedback might weaken the link between learning goals and the behavior of asking students for feedback.

### Present Research

**FIGURE 1:**
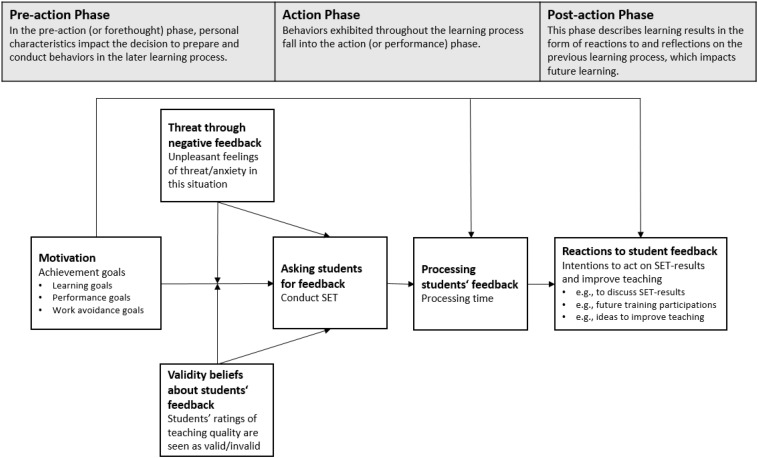
Proposed model for the voluntary usage of SETs as a learning situation.

We aim to shed light on whether and how instructors’ achievement goals impact learning from SETs during different phases of self-regulatory learning (pre-action phase = decision to use and conduct SETs; action phase = processing of SETs; post-action phase = intentions to act on SET-results and improve future teaching; see Figure 1) in a longitudinal online study. Regarding the pre-action phase, we assumed that learning approach, learning avoidance, and performance approach goals positively predict whether instructors conduct voluntary SETs. In contrast, we assumed that performance avoidance and work avoidance goals negatively predict whether university instructors conduct SETs voluntarily. Furthermore, we expected that the strength of the association between learning goals and the usage of SETs is moderated by beliefs in the validity of SETs and the degree to which negative feedback is experienced as threatening. More precisely, the more instructors perceive SETs as valid measures of teaching quality and the less they feel threatened by negative feedback in general, the stronger the associations should be. Besides these moderation effects, we also assumed that beliefs in the validity of SETs positively predict, and generally experiencing threat after negative feedback negatively predict voluntary use of SETs directly.

Focusing on the later learning phases, we assumed that both learning approach and learning avoidance goals positively predict the time spent processing student feedback (as an objective measure of effort) in the action phase as well as intentions to act on SET-results and improve future teaching in the post-action phase. In contrast, we expected work avoidance goals to negatively predict these variables. We also expected that the time spent processing student feedback would mediate the associations between learning approach/avoidance and work avoidance goals and the postulated post-action phase outcome variables (intentions to act on SET-results and improve teaching). As differences in the amount and content of feedback might also impact processing time and intentions to act on SET results and improve future teaching, this should be controlled for in studies in natural settings.

To ensure that the observed relations are robust for differences in the quantity and quality of SETs, we considered teaching quality, number of students, and number of questions in the SETs as control variables. In an experimental study, instructors believed in the validity and trustworthiness of the results to a stronger extent if the participation rate of students in the processed SET-results was higher (Nowakowski and Hannover, 2015). Consequently, instructors might correctly interpret students’ feedback as invalid information if the participation rate is very low, and thereby spend less time on it. In addition, SETs deliver more information to process if the number of students who answer the evaluation survey is higher (e.g., due to more open qualitative comments by students), or if the number of questions within the evaluation survey increases (e.g., if instructors add their own questions). We assume that the number of students is positively associated with the time it takes to process student feedback and both intentions regarding SET-results (as validity and quantity of feedback increases with the number of students). Moreover, positive and negative feedback can be beneficial for subsequent learning (Hattie and Timperley, 2007). Instructors could react to poor ratings of teaching quality in different ways (e.g., avoid processing the results to maintain self-worth, or examining the results more closely as step toward improvement). As it is not clear how teaching quality affects the later learning process, we explore the associations of this control variable with the subsequent steps in the learning process (for processing time as well as intentions to act on SET-results and improve teaching).

## Materials and Methods

We conducted a longitudinal study^2^ to investigate our research questions. In this study, we used data from an online open-access website^3^ that allowed instructors to administer SETs for their courses. We used a mixture of self-reports (e.g., achievement goals, intentions to act on SET-results and improve future teaching) and objective behavioral data (e.g., conducting voluntary SETs with the online tool, time spent processing the SETs) to investigate how achievement goals impact the different phases of self-regulated learning from SETs.

### Procedure

The open-access platform was designed for the purposes of this study(Janke et al., 2020) and advertised at 21 higher education institutions in Germany and Austria through direct mail inquiries (total reach = 18,084 instructors). The professional contexts in higher education institutions in Germany and Austria share many (structural) similarities^4^. The participation in this study was voluntary for all instructors. After registering, all participants were asked to answer a baseline questionnaire. After finishing this baseline questionnaire, participants were prompted to register their courses for SETs within the online platform. Instructors voluntarily used this option. The instructors were allowed to evaluate as many courses as they wanted and could also use the evaluation tool after the first semester of study participation. In contrast to our study design, SETs in German-speaking countries are typically administered by the higher education institutions where participation is usually mandatory, and these mandatory SETs are often not linked to immediate consequences within the higher education institution. However, SETs are an important feedback tool, and are relevant for later job applications and tenure. Nevertheless, next to mandatory SETs, instructors are allowed to additionally conduct voluntary SETs that can also be used for later job applications. To administer the SETs themselves, we used a well-validated scale (SEEQ; Marsh, 1982) in our study. Access to the questionnaire was given to the students via codes that were either distributed via mail or printed out by the instructors. After the evaluation, the SET-results were presented to the instructors online in our study. We advised them to process the results of the SETs for the first time when they had sufficient time to so. Immediately after processing the SET-results online, instructors were invited to answer a short questionnaire on their intentions regarding SET-results (intentions to act and intentions to improve teaching). After participating in the short questionnaire or after the study participation in the longitudinal study ended, instructors additionally received the SET-results as PDF files via E-mail for personal storage and future applications. The data of the two questionnaires and the anonymized data derived from the platform was matched using electronically generated codes. We assured the participants that their answers would remain confidential and would only be used for scientific purposes. The instructors received incentives for their participation in every questionnaire (choice between a direct monetary reward or a donation to a charity; 5 Euro [approx. 6 US $ at that time] was offered per questionnaire).

### Sample

Overall, 796 instructors (412 male, 372 female, 12 diverse) registered for the online platform by the end of March 2020^5^ (response rate around 4%), while 458 of these instructors finished the first questionnaire (participation rate: 57%). We deleted the data of 16 instructors who did not assert that we could use their data for research purposes, and excluded one instructor who had no code for matching the data. We excluded another 34 participants who did not report a teaching commitment for at least one course within the semester of study participation from our analyses, as they did not have the opportunity to evaluate a course, which was a requirement for the study.

This resulted in a net sample of 407 Austrian and German higher education instructors (52.6% male, 46.4% female, 1.0% diverse; average age: 38.60 years, *Min* = 20 years, *Max* = 75 years, *SD* = 10.21 years) with baseline data and teaching commitment. The instructors had an average of 8.91 years of teaching experience (*Min* = 0, *Max* = 42, *SD* = 8.38, 0.2% missing data). They were employed in a wide array of disciplines, mostly in universities (94.8%) but also in universities of applied sciences (2.0%), universities of cooperative education (0.2%), colleges of arts and music (1.7%), and colleges of public administration (0.7%, 0.5% missing data). The instructors reported their highest level of education (1.0% with bachelor degree, 39.8% with masters’ degree, 44% with Ph.D, 15.2% with habilitation, no missing data). As one of the formal qualifications to teach in German or Austrian higher education institutions, instructors need to have a degree higher than the students. The sample consisted of higher education instructors in diverse employment situations including 67.1% in temporary positions and 27.5% in permanent positions (5.4% missing data); 33.8% doctoral candidates (32.4% academic staff pursuing a Ph.D, 0.7% master graduates with scholarships pursuing a Ph.D, 0.7% masters graduates pursuing a Ph.D. next to working outside of higher education institutions), 28.5% post-docs (academic staff pursuing a habilitation), 18.4% professors (2.2% junior/assistant professors, 16.2% full professors), 17% of the sample reporting additional teaching assignments for one semester (which can be granted to internal and external individuals in higher education institutions with at least a master’s degree). The sample of 407 instructors reported to spend on average 36.3% of their working time on teaching, 41.7% of their time on research and 21.8% of their time on administration. The reported percentage of working time spent on teaching-related activities did not differ remarkably across doctoral candidates, post-docs, and professors (32.8 to 34.3%). It is important to note that doctoral candidates are predominantly members of the academic staff in Germany and Austria, and therefore take on tasks in research, teaching, and administration comparable to other instructors in higher education.

Out of the net sample of 407 instructors, 152 instructors conducted at least one voluntary evaluation within the same semester. These instructors participated with 171 courses overall (*N* = 1672 students, 30.2% male, 61.5% female, 3.9% missing data, mainly bachelor students with 36.5% in their first year, 26.6% in their second year, 18.0% in their third year of study, 14.5% in later study years). In Mann-Whitney-*U*-Tests, the subsample of 152 instructors (50.0% male, 49.3% female, 0.7% diverse; average age: 38.68 years, *Min* = 20 years, *Max* = 65 years, *SD* = 10.24 years) who conducted at least one SET did not differ significantly in age (*U* = 19097.50, *Z* = –0.114, *p* = 0.909), academic status (*U* = 18774.50, *Z* = –0.574, *p* = 0.566), or teaching experience (*U* = 18659.50, *Z* = –0.565, *p* = 0.572) compared to instructors who did not conduct SET(s).

### Measures

#### Baseline Questionnaire

##### Achievement Goals in Teaching

Higher education instructors reported their current teaching-related achievement goals with a well-validated questionnaire (Daumiller et al., 2019). All items used the item stem “In my current teaching activities…”. We assessed instructors’ learning approach (e.g., “…I want to constantly improve my competences”; ω = 0.93), learning avoidance (e.g., “…it is important to me to avoid having my competences not develop further”; ω = 0.90), performance (appearance) approach (e.g., “…I want to be perceived as competent”; ω = 0.90), performance (appearance) avoidance (e.g., “…I want to avoid being perceived as incompetent”; ω = 0.94), and work avoidance goals (e.g., “…I want to have as little to do as possible”; ω = 0.95) with four items each^6^. We focus on the appearance component of performance goals and thereby use the terms appearance approach and appearance avoidance goals in the manuscript from here on. All items were answered on Likert-type scales ranging from 1 (*do not agree at all*) to 8 (*agree completely*). We used confirmatory factor analyses to ensure the reliability and structure of these five goal types (χ^2^ = 453.5, CFI = 0.94, TLI = 0.92, RMSEA = 0.07, SRMR = 0.05).

##### Beliefs in the Validity of SETs

We used a slightly adapted scale measuring beliefs in the validity of SETs (Nowakowski and Hannover, 2015) to assess how strongly instructors believe that students can capture teaching quality in general (e.g., “I believe that students are able to realistically assess the teaching quality of a course.”, ω = 0.84). All five items were answered on Likert-type scales ranging from 1 (*do not agree at all*) to 5 (*agree completely*). The five-item scale contained four positively and one negatively worded item (the latter item was recoded when calculating the average score across the items). High scores represent positive beliefs in the validity of SETs and imply that instructors are convinced that student evaluations are valid indicators of teaching quality. Confirmatory factor analyses also speak to the reliability and structure of this scale (χ^2^ = 454.6, CFI = 0.99, TLI = 0.98, RMSEA = 0.04, SRMR = 0.02).

##### General Experienced Threat Through Negative Feedback

To assess instructors’ general experienced threat through negative feedback, we used a threat subscale of a well-validated questionnaire (Gaab, 2009) that refers to threat experience within concrete situations. The concrete situation needs to be described before displaying the items. We specified the concrete situation by asking the instructors how they feel when they receive negative feedback about their teaching from students or colleagues with four items (e.g., “Negative feedback is very unpleasant for me.;” ω = 0.76). All four were measured with a Likert-type scale ranging from 1 (*completely wrong*) to 6 (*entirely true*). The four-item scale contained two positively and two negatively worded items (the latter items were recoded before calculating the average score). High scores represent stronger experienced threat through negative feedback. Confirmatory factor analyses further confirm the reliability and structure of the scale on threat through negative feedback (χ^2^ = 305.1, CFI = 0.99, TLI = 0.95, RMSEA = 0.08, SRMR = 0.01).

#### Behavioral Data (Derived From the SET-Platform)

##### Conducting Voluntary Sets

To assess whether instructors conducted at least one SET within one course within 6 months after answering the baseline questionnaire, this information was coded as a dichotomous variable ranging from 0 (no course evaluated) to 1 (at least one course evaluated). A total of 152 of 407 instructors conducted an evaluation of at least one course with the provided online tool, and thereby 37% of the instructors that reported a current teaching commitment.

##### Processing Time Regarding SET-Results

The processing time, more precisely, the time that instructors had left the evaluation results open online (in the displayed tab in their browser) before starting the second questionnaire, was tracked as log data within the system measured in milliseconds. This measure accurately indicates the time that the SET-results^7^ were viewed for. To facilitate interpretation of the time stamps, we converted the data from milliseconds into minutes. Furthermore, we identified outliers which could indicate that instructors had left the tab window open while being away from their desk or doing other tasks. Specifically, we replaced extremely high processing times (above 2 h) for 19 participants with -99 (*missing values*). Participants that did not process the SET-results online before data was retrieved were treated as missing data ‘-99’. The processing times ranged between 0.15 and 31.17 mins for processing the results online.

#### Second Questionnaire (Filled Out Immediately After Processing the SET)

##### Intentions to Act

We used a slightly adapted German self-report scale (Nowakowski and Hannover, 2015) as a *quantitative measure* for the *intentions to act* on SET-results. The self-report scale captures the intentions to discuss the concrete SET-results with students and colleagues, to make changes in future courses, and participate in didactical trainings with six items (e.g., “Based on this feedback I will make concrete changes to my course.”; ω = 0.65). All six items were answered on Likert-type scales ranging from 1 (*do not agree at all*) to 5 (*agree completely*). Confirmatory factor analyses further confirm the reliability and structure of the quantitative measure of intentions to act on SETs (χ^2^ = 97.1, CFI = 0.90, TLI = 0.83, RMSEA = 0.08, SRMR = 0.06). The answers to one single item were recoded, so that high scores consistently represent stronger intentions to act on the SET-results.

##### Intentions to Improve Teaching

We used one open-ended question as a *qualitative measure* for *intentions to improve teaching*. Specifically, we asked the instructors “How will you improve your course in the next semester based on the provided feedback? Please make suggestions”. Two independent raters assessed how many distinct concrete ideas for improving their teaching the instructors reported within their answers. Precisely formulated ideas for concrete changes to improve future teaching and globally formulated ideas were counted (coding options: 0 = no ideas formulated; –99 = missing values due to non-participation in the second questionnaire). If instructors tried to reach one purpose by several precise changes, all diverse purposes were counted. The two raters agreed in 87.6% of their judgments (Cohens κ = 0.93). We used the average score across both ratings regarding the absolute number of distinct concrete ideas for future improvements of teaching as a qualitative measure for the intentions to improve teaching. High scores represent stronger reported intentions to improve teaching.

#### Control Variables

##### Employment Situation/Permanent Position

The instructors reported whether they were employed in a temporary (0) or permanent contract (1).

##### Academic Status

The instructors reported their academic status as doctoral candidates (1), post-docs (2), or professors (3) in a close ended question. Three dichotomous variables regarding the academic status were entered as control variables in the later analyses (‘0’ concrete status not applicable, ‘1’ concrete status applicable).

##### Low Teaching Quality

For teaching quality, we used a single item of the SEEQ (Marsh, 1982, 1984) that is meant to indicate overall teaching quality. To elaborate, the students were asked to assign an overall grade to the course ranging from 1 (*very good*) to 5 (*poorly*) with low grades indicating good teaching (German grading system). The German grading system was applied, as the students are familiar with this system. However, this implies that high scores represent low teaching quality within a course. As instructors were free to evaluate multiple courses—we used the average score across the SET(s) instructors conducted within one semester after the baseline questionnaire before answering the short questionnaire.

##### Number of Students

The average score of students participating in the first SET(s) was calculated within one semester before answering the short questionnaire, which was used for further analyses.

##### Number of Courses

The number of evaluated courses within one semester before answering the short questionnaire was used for further analyses to control for differences in the quantity of students’ feedback.

##### Number of Additional Questions

As instructors could enter additional questions to the student survey within the online evaluation system, we counted the number of additional questions per instructor for the included courses to control for different amounts of information instructors received within their SET-results.

### Analyses

Not all instructors who were theoretically able to use the platform for evaluations (indicated by reported teaching commitments) chose to conduct SET(s), as this was a voluntary option. For this reason, we carried out separate analyses for predicting the initiation of learning from SETs by using the platform to conduct SET(s) (pre-action phase of self-regulated learning) with the full sample and for the later learning process (action and post-action phase of self-regulated learning) with the reduced sample. We conducted structural equation models for our main analyses with manifest scores using Mplus Version 8.5 (Muthén and Muthén, 2017). We used the maximum likelihood estimator with robust standard errors (MLR) and the weighted least squares means and variance (WLSMV)-adjusted estimator (for analyses with categorical outcomes), which are robust to multivariate non-normality because our data violated the assumption of normal distribution in Kolmogorov-Smirnov tests for all variables (with the exception of the intentions to act). We log transformed the processing time because the time data violated the assumption of normal distribution. We report standardized parameter estimates for better interpretability of all findings. Standardized parameters reflect how many standard deviations an outcome variable changes per standard deviation increase in the predictor variable. For regression coefficients, when we had directed hypotheses, we reported one-tailed levels of significance.

#### Missing Values

We had no missing values on any variables assessed in the baseline questionnaire. However, out of the 152 instructors who conducted SET(s), only 132 also answered the short questionnaire (13.1% missing data regarding intentions to act on SET-results and improve teaching). As we coded the processing time for 19 participants as missing data due to outliers with very high viewing times (see above), we had in total 17.1% missing data regarding processing time. Finally, some participants had missing values on the indicator for teaching quality for all students that had participated in the SETs (1.3% missing data). We used a full information maximum likelihood approach (FIML) to handle missing data and include all available information for model estimations. This method increases the power of the data analysis and reduces the impact of bias due to missing data (Enders, 2010).

#### Pre-Analyses

To ensure the comparability of the diverse sample subgroups regarding their employment situation (temporary or permanent position) and academic status (doctoral candidates, post-docs, and professors) multivariate ANOVAs were conducted. The multivariate ANOVAs are reported in the “Results” section.

#### Pre-action Phase to Action Phase of Self-Regulated Learning (*N* = 407)

We estimated bivariate and multivariate models to assess whether achievement goals predicted if the instructors voluntarily conducted SET(s) using the net sample (*N* = 407). In these models, latent factors were estimated for the predictor variables (achievement goals, beliefs in the validity of SETs, and general experienced threat through negative feedback). In the first multivariate model, we regressed whether instructors had conducted SET(s) voluntarily as a dichotomous measure on instructors’ achievement goals and the two moderator variables (main effects). In the subsequent model, we added the control variables (employment situation and academic status) to test for the robustness of the results. In both multivariate models, we allowed for correlations between all predictor variables. In addition, we allowed for residual correlations of items with similar wordings between the approach and avoidance items of achievement goals. We included residual correlations between negatively worded items of experienced threat.

Considering categorical outcomes using the WLSMV-adjusted estimator was only possible in manifest interaction analyses, we calculated manifest models to examine, whether beliefs in the validity of SETs and general experienced threat through negative feedback moderated the relationship between learning (approach and avoidance) goals and the behavior of conducting voluntary SETs. The moderation models were estimated for both moderators and learning goals separately (resulting in four moderation models). We allowed for correlations between all predictor variables (including interaction terms) in all interaction models. The moderation models were fully saturated (Raykov et al., 2013).

#### Pre-action to Action and Post-action Phase of Self-Regulated Learning (*N* = 152)

We estimated a latent structural equation model to test the mediation hypotheses regarding intentions to act and improve teaching based on the subsample (*N* = 152) for each achievement goal type, if correlational results suggested a possible mediation effect. In the mediation models, we estimated the specified latent factors for the considered achievement goals and the variable interaction to act on the SET-results on the manifest item scores per construct. More precisely, we regressed both learning outcomes of the post-action phase (intentions to act and improve teaching) on the relevant achievement goal of the pre-action phase (learning approach or avoidance goals or work avoidance goals), on the indicator for the action phase (processing time), and on four control variables (low teaching quality, number of students, number of courses, additional questions) to control for quantitative and qualitative differences in the feedback instructors received within the SET-results. Additionally, processing time was regressed on the achievement goals and the above mentioned control variables. Indirect effects of the single achievement goals via processing time on both outcome variables were calculated in these models. We allowed for correlations of the outcome variables (intentions to act and improve teaching) and correlations between all predictor variables (achievement goals and control variables).

#### Model Fit

Because χ^2^ is overly sensitive for small deviations in large samples (Chen, 2007; for an overview see Putnick and Bornstein, 2016), absolute fit indices are reported. We used the Comparative Fit Index (CFI), Tucker Lewis Index (TLI), Root Mean Square Error of Approximation (RMSEA), and Standardized Root Mean Square Residual (SRMR) as fit indices to determine the model fit. Absolute fit indices for CFI and TLI values greater than 0.90 (Hu and Bentler, 1999); and RMSEA values below 0.08, and SRMR values below 0.10 constitute an acceptable fit (for a comparison, Schermelleh-Engel et al., 2003).

## Results

The descriptive statistics and correlations are reported in Table 1. Multivariate ANOVAs overall revealed statistically significant mean differences in the model-relevant predictor variables of the pre-action phase that were assessed in the baseline questionnaire (*N* = 407) regarding the instructors’ employment situation (temporary or permanent position; Wilks λ = 0.94, *F*[7,377] = 3.70, *p* = 0.001), and their academic status (doctoral candidates, post-docs, and professors; Wilks λ = 0.93, *F*[14,634] = 1.81, *p* = 0.033). Instructors employed at temporary or permanent positions differed statistically significantly in their general experienced threat through negative feedback (*F*[1,383] = 14.71, *p* < 0.001). The strength of work avoidance goals differed statistically significant for instructors with different academic statuses (*F*[2,323] = 5.70, *p* = 0.004).

**TABLE 1:** Descriptive statistics for all variables and correlations.

	*Min/Max*	*M(SD)*	[1]	[2]	[3]	[4]	[5]	[6]	[7]	[8]	[9]	[10]	[11]	[12]	[13]	[14]
[1] Learning approach goals	1/8	6.96 (1.16) /6.92 (1.14)		**0.52** <0.001	**0.22** <0.001	0.03 0.567	**–0.27** <0.001	**0.16** <0.001	0.06 0.249	–0.03 0.601	–	–	–	–	–	–
[2] Learning avoidance goals	1/8	6.18 (1.74) /6.43 (1.60)	**0.44** <0.001		**0.18** <0.001	**0.25** <0.001	**–0.17** <0.001	**0.11** 0.010	0.06 0.222	**0.14** 0.027	–	–	–	–	–	–
[3] Appearance approach goals	1/8	6.01 (1.48) /6.12 (1.37)	**0.21** 0.008	0.08 0.365		**0.59** <0.001	**0.10** 0.035	**0.15** 0.001	**0.19** <0.001	0.07 0.286	–	–	–	–	–	–
[4] Appearance avoidance goals	1/8	6.03 (1.94) /6.07 (1.87)	–0.03 0.712	**0.19** 0.029	**0.55** <0.001		**0.21** <0.001	0.04 0.382	**0.26** <0.001	0.02 0.774	–	–	–	–	–	–
[5] Work avoidance goals	1/8	2.80 (1.77) /2.78 (1.75)	**–0.41** <0.001	**–0.27** 0.002	0.13 0.076	**0.19** 0.007		–0.07 0.141	0.02 0.763	–0.01 0.870	–	–	–	–	–	–
[6] Validity beliefs regarding SETs	1/5	3.55 (0.73) /3.58 (0.75)	0.17 0.157	0.17 0.075	**0.18** 0.021	0.14 0.075	–0.11 0.231		**–0.12** 0.016	0.04 0.559	–	–	–	–	–	–
[7] Experienced threat	1/6	2.92 (1.06) /2.97 (1.07)	–0.01 0.849	0.03 0.720	**0.21** 0.011	**0.33** <0.001	0.08 0.333	–0.08 0.302		0.05 0.456	–	–	–	–	–	–
[8] Conducting voluntary SET(s)	0/1	n/a	–	–	–	–	–	–	–		–	–	–	–	–	–
[9] Processing time in minutes	0/31	4.26 (4.21)	0.07 0.330	0.10 0.218	0.01 0.952	**0.20** 0.007	–0.02 0.845	0.05 0.529	0.08 0.359	–		–	–	–	–	–
[10] Intentions to act	1/5	2.94 (0.78)	**0.33** <0.001	**0.20** 0.018	0.07 0.368	–0.09 0.307	–0.13 0.131	0.01 0.918	–0.13 0.102	–	0.15 0.054		–	–	–	–
[11] Intentions to improve teaching”	0/5	1.34 (1.08)	**0.23** 0.006	–0.01 0.942	0.13 0.066	0.02 0.781	0.01 0.964	0.07 0.408	–0.02 0.751	–	**0.22** 0.014	**0.45** <0.001		–	–	–
[12] Low teaching quality	1/5	1.87 (0.56)	–0.17 0.082	–0.09 0.298	**–0.17** 0.022	–0.04 0.660	0.06 0.465	**–0.19** 0.010	–0.03 0.711	–	0.15 0.054	0.11 0.224	–0.01 0.897		–	–
[13] Number of courses	1/4	1.12 (0.43)	0.02 0.775	0.01 0.874	0.04 0.695	0.06 0.294	–0.00 0.988	0.02 0.874	0.11 0.238	–	0.02 0.740	–0.01 0.875	–0.15 0.011	0.03 0.656		–
[14] Number of students	1/45	10.05 (8.27)	0.090 0.272	0.05 0.595	–0.05 0.599	–0.04 0.623	–0.06 0.531	0.10 0.123	**–0.20** 0.003	–	**0.39** <0.001	**0.24** <0.001	**0.29** <0.001	0.01 0.898	**–0.10** 0.012	
[15] Additional questions	0/15	0.91 (2.20)	–0.03 0.608	0.02 0.767	**–0.09** 0.318	–0.04 0.633	0.02 0.776	0.08 0.280	**–0.09** 0.259	–	0.19 0.058	0.04 0.622	**0.20** 0.007	–0.07 0.260	–0.02 0.514	0.25 0.070

*Min = Minimal (rounded); Max = Maximum (rounded); M, Mean; SD, Standard deviation. Descriptive characteristics are reported for the fullest available sample (*N* = 407 for [1-8]; N = 152 for [9-15]). After the dash [/], mean values and standard deviations for the subsample of instructors who conducted SET(s) are additionally reported (for the constructs [1] to [7]). The zero-order correlations are derived from two saturated base models in which undirected paths between all included variables were freed. The correlations under the diagonal are for the subsample (*N* = 152) of instructors who evaluated (estimator: MLR), while the correlations above the diagonal are for the net sample of instructors with teaching commitments from the baseline questionnaire (*N* = 407). The zero-order correlations are derived from two saturated base models in which undirected paths between all variables were freed. Significant correlations (*p* < 0.05) are are printed in boldface and two-tailed levels of significance are reported under the correlations.*

Overall, the analyses revealed no statistically significant mean differences in the model-relevant predictor variables that were assessed within the SET-tool and short questionnaire for the subsamples of instructors (*N* = 152), who conducted SET(s) regarding their employment situation (Wilks λ = 0.91, *F*[7,107] = 1.57, *p* = 0.153) or their academic status (Wilks λ = 0.80, *F*[14,176] = 1.45, *p* = 0.136). See Supplementary Material 3 for the subgroup specific descriptive statistics.

Because multivariate ANOVAs partly revealed significant group differences in the predictor variables, we additionally controlled for instructors’ employment situation and/or academic status in the following structural equation models that included either threat through negative feedback or work avoidance goals.

### Pre-action Phase to Action Phase of Self-Regulated Learning (*N* = 407)

As expected, we found positive associations between learning avoidance goals and voluntarily conducted SET(s) in our sample of higher education instructors (see Table 1). In multivariate analyses, this association was robust even when we controlled for the other achievement goals (see Table 2, Model 1) as well as further control variables regarding the employment situation and academic status (see Table 2, Model 2). In addition, our results confirmed our hypotheses concerning associations of appearance (approach/avoidance) goals and voluntarily conducted SET(s) in the multivariate analyses. However, we found no statistically significant associations for the learning approach and work avoidance goals or the moderator variables, neither in bivariate nor in multivariate analyses. Nevertheless, the bivariate associations of conducting voluntary SET(s) and work avoidance goals pointed descriptively in the expected direction. Achievement goals and the moderators only explained a significant proportion of the variance in later voluntarily conducted SET(s) in the multivariate model that controlled for the instructors’ employment situation and academic status (*R^2^* = 0.09, *p* = 0.041). Moreover, we did not find any statistically significant moderation effects in the additional models on the supposedly relevant interactions of beliefs in the validity of SETs and general experienced threat in light of negative feedback with learning goals (see Table 3).

**TABLE 2:** Results of the latent SEMs for associations with later voluntary conducted SET(s).

	Bivariate Models			Multivariate Model 1			Multivariate Model 2		
			
	β	*SE*	*p*	β	*SE*	*p*	β	*SE*	*p*
Learning approach goals	−0.04	0.06	0.705	−0.27	0.09	0.998	−0.27	0.10	0.998
Learning avoidance goals	**0.15**	0.07	0.013	**0.31**	0.10	0.001	**0.31**	0.10	0.001
Appearance approach goals	0.08	0.07	0.131	**0.17**	0.10	0.038	**0.16**	0.10	0.048
Appearance avoidance goals	0.02	0.07	0.614	**−0.18**	0.10	0.035	**−0.19**	0.10	0.026
Work avoidance goals	−0.01	0.07	0.429	−0.01	0.07	0.454	0.00	0.07	0.504
Validity beliefs	0.05	0.07	0.261	0.04	0.07	0.302	0.04	0.07	0.293
Experienced threat	0.05	0.07	0.754	0.05	0.08	0.742	0.08	0.09	0.811
Permanent position (CV)	0.04	0.07	0.577	–	–	–	0.12	0.09	0.193
Doctoral candidates (CV)	0.04	0.06	0.574	–	–	–	0.07	0.09	0.462
Post-docs (CV)	0.01	0.06	0.878	–	–	–	0.02	0.09	0.791
Professors (CV)	−0.05	0.06	0.424	–	–	–	**−0.10**	0.09	0.238
*R*^2^	n/a			*R^2^* = *0.07*, *p* = 0.064			*R^2^* = *0.08*, *p* = 0.043		

**N* = 407; β = standardized regression coefficient; *SE* = standard error; *p* = one-tailed level of significance (two-tailed level of significance for control variables; CV = control variable. Significant effects (*p* < 0.05) are printed in boldface. The achievement goals and investigated moderators (experienced threat and validity beliefs) are modeled as latent variables in all reported models. The correlations of predictor variables and error variances varied between –0.29 and 0.63 in Model 1; and between –0.45 and 0.62 in Model 2. The model fit the data sufficiently well (for Model 1: CFI = 0.90, TLI = 0.89, RMSEA = 0.04, SRMR = 0.04; for Model 2: CFI = 0.95, TLI = 0.94, RMSEA = 0.03, SRMR = 0.04).*

**TABLE 3:** Results of the manifest moderation analyses.

	Model 1			Model 2			Model 3			Model 4		
				
	β	*SE*	*p*	β	*SE*	*p*	β	*SE*	*p*	β	*SE*	*p*
Learning approach goals	–0.04	0.07	0.710	–0.04	0.06	0.734	–	–	–	–	–	–
Learning avoidance goals	–	–	–	–	–	–	**0.15**	0.06	0.011	**0.14**	0.06	0.017
Validity beliefs	0.04	0.06	0.252	–	–	–	0.03	0.06	0.329	–	–	–
Experienced threat	–	–	–	0.06	0.06	0.815	–	–	–	0.04	0.06	0.729
Interaction	0.00	0.06	0.492	–0.08	0.06	0.097	0.07	0.06	0.137	–0.02	0.06	0.405
*R*^2^	*R^2^* = *0.00*, *p* = 0.667			*R^2^* = *0.01*, *p* = 0.431			*R^2^* = *0.03*, *p* = 0.217			*R^2^* = *0.02*, *p* = 0.245		

**N* = 407; β = standardized regression coefficient; *SE* = standard error; *p* = one-tailed level of significance. The reported interaction effect always describes the interaction of the predictor variables that are contained in the model. Significant effects (*p* < 0.05) are printed in boldface. In the moderation models, we allowed for correlations of predictor variables, which varied between –0.38 and 0.16 in Model 1, –0.04 and 0.09 in Model 2, –0.15 and 0.11 in Model 3, –0.12 and 0.06 in Model 4.*

### Pre-action Phase to Action and Post-action Phase of Self-Regulated Learning (*N* = 152)

**FIGURE 2:**
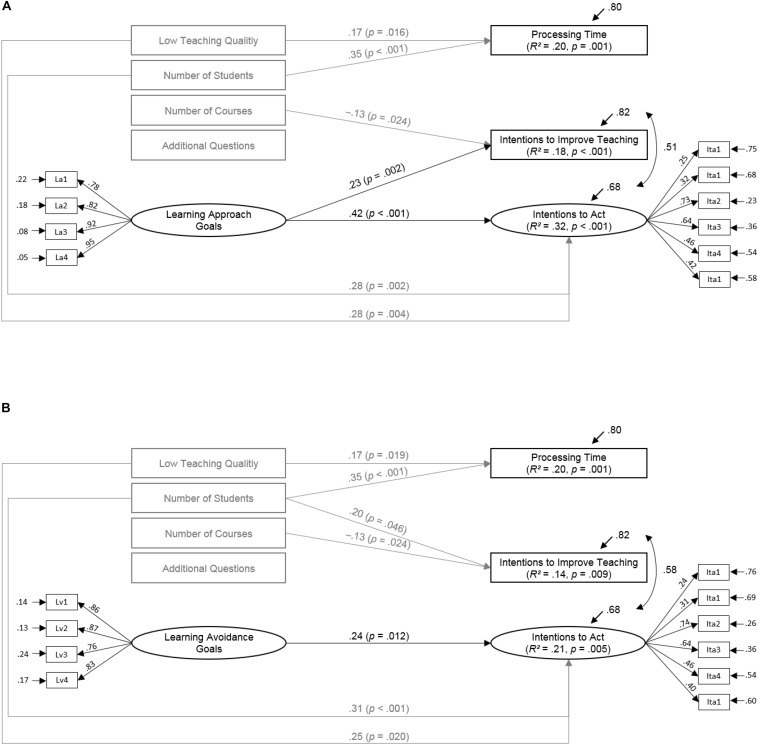
Results of the mediation models for the associations between learning approach/avoidance goals and intentions to act on SET-results and improve teaching via processing time (*N* = 152). Only statistically significant paths are depicted (*p* < 0.05). One-tailed significance levels are reported for directed hypotheses (depicted in black); two-tailed levels of significant are reported for the associations with control variables (depicted in gray). The correlations between the predictor variables varied between –0.36 and 0.74 in Model **(A)** and between –0.10 and.25 in Model **(B)**.

Figure 2A for learning approach goals and Figure 2B for learning avoidance goals depict the significant standardized path coefficients derived from the structural equation models on later learning phases. The models adequately fit the data (for learning approach goals: CFI = 0.93, TLI = 0.90, RMSEA = 0.06, SRMR = 0.05; for learning avoidance goals: CFI = 0.97, TLI = 0.96, RMSEA = 0.03, SRMR = 0.05). Since work avoidance goals were not significantly associated with processing time, intentions to act, or intentions improve teaching (see Table 1), we did not conduct mediation analyses on this goal type. The multivariate mediation models for learning approach and avoidance goals explained substantial amounts of variance for indicators of the action (20% of processing time) and post-action phases (21 to 32% of intentions to act and 14 to 18% of intentions to improve teaching).

Learning (approach and avoidance) goals were not statistically significantly associated with processing time in the multivariate or bivariate models. As expected, learning approach and avoidance goals positively predicted later reported intentions to act on the SET-results in bivariate and multivariate analyses. However, only learning approach goals were positively associated with the number of intentions to improve teaching. The bivariate positive relation of processing time and intentions to improve teaching did not emerge when controlling for effects of the learning goals and further control variables in the multivariate models. Moreover, we found no indirect effects of the suspected achievement goals via processing time on intentions regarding SET-results (neither for intentions to act nor for intentions to improve teaching). The indirect link of learning goals and intentions to act on the SET-results could not be found for learning approach goals (β = –0.00, *SE* = 0.01, *p* = 0.604) or learning avoidance goals (β = –0.00, *SE* = 0.01, *p* < 0.567). Congruently, no indirect effects of the learning goals on the intentions to improve teaching were statistically significant (learning approach goals: β = 0.01, *SE* = 0.01, *p* < 0.204; learning avoidance goals: β = 0.01, *SE* = 0.01, *p* = 0.164).

Interestingly, teaching quality was positively associated with processing time and intentions to act on the SET-results (in both models). This means that the worse the teaching quality was rated, the more time it took instructors to process the results and the higher their intentions were to act on the results they processed. In addition, the more students participated in the SET, the more time instructors needed to process the results (in both models), the stronger the intentions to act on the SET-results were (in both models), and the higher the number of intentions to improve future teaching (only in one model). Moreover, the number of courses instructors evaluated was negatively associated with the number of intentions to improve teaching (in both models).

## Discussion

In our longitudinal field study, we aimed to investigate whether and how achievement goals predict self-regulated learning with SETs within university instructors. We found that especially learning avoidance goals, but also appearance approach and avoidance goals, predicted the instructors’ behavior to voluntarily conduct SET(s). We found no effects for the other achievement goals or any moderation processes through beliefs in the validity of SETs or experienced threat concerning negative feedback on the behavior to voluntarily conduct SET(s). In contrast, learning approach goals predicted later self-reported intentions to act on SET-results and improve future teaching, while learning avoidance goals were only associated with later reported intentions to act on SET-results. Contrary to our initial assumptions, the positive associations of learning approach/avoidance goals and instructors’ intentions were not mediated by their processing time of SET-results.

### Theoretical Implications

Our study advances research on learning from SET-results, as we proposed a model that explains what motivates instructors to voluntarily use student evaluations of teaching and learn from these results. Such a model is desperately needed, as the impact of SETs depends on instructors’ openness to student feedback and their willingness to engage with the evaluation results (Kember et al., 2002). In line with models of self-regulated learning, we found that instructors’ achievement goals predicted necessary learning steps during the pre-action phase (conducting voluntary SETs), the action phase (processing of SETs), and the post-action phase (intentions to act on SET-results and improve teaching). We do not claim that our theoretical framework on motivated usage of SETs and processing exhaustingly describes all processes that lead instructors to conduct and learn from SETs, as substantial proportions of variance on the criteria are not yet explained. However, we provide a foundation for further research on the subject matter. In this regard, our results underline the crucial importance of learning goals as facilitators of self-regulated learning in- and outside of higher education (Nitsche et al., 2013; Diethert et al., 2015; Hein et al., 2019, 2020; Daumiller et al., 2021c).

From a methodological perspective, we contribute to the literature by using behavioral measures such as the actual use of voluntary SETs and processing time to investigate how instructors use SET(s). This results in more realistic estimations of the predictive power of achievement goals than when only relying on self-report measures. This advancement, however, comes with the caveat that we only found small associations of achievement goals with the behavioral indicator of conducting voluntary SET(s), and no associations with processing time (except for an unexpected correlation with appearance avoidance goals). One possible explanation for this pattern of results is that processing time itself might be limited in its reliability and validity, as there could be multiple reasons that lead instructors to keep the tab with the SET-results open (aside from looking at them). In contrast, we found empirical evidence for associations between achievement goals and the voluntary use of SETs in our study. However, it is noteworthy that the amount of explained variance in the objective outcome variable, voluntary conducted SET(s), was only significant when controlling for instructors’ employment situation and academic status. Thereby, the practical relevance of the associations in the pre-action phase is unclear and should be further investigated in future studies. To find even a small association of teaching-related achievement goals and voluntarily conducting SET(s) as a behavioral measure, is highly interesting. To this end, the amount of explained variance in conducting voluntary SET(s) having not been significant in the model including the achievement goals without further control variables as predictors may have been due to a very small effect. The sample size might have limited the power to detect such a small effect. Moreover, the constructs are operationalized on different levels, because we assessed general teaching-related achievement goals instead of concrete SET-related goals.

Our design allows for temporal ordering of most of the variables (achievement goals, voluntary conducted SETs, processing time for SETs-results and intentions regarding SET-results) and thereby prospective analyses. This helps us to gather an even more cohesive picture about the learning process and to distinguish different phases in line with models of self-regulated learning. The depicted process underlines the validity of such models. Without deciding to use SETs and conducting them, instructors have no chance to interpret the results or to form intentions to act on SET-results and improve future teaching. Despite a lack of predictive power when additionally considering achievement goals, we found that processing time was indeed predictive of the number of derived ideas to further one’s teaching in bivariate analyses. This clearly speaks to the notion that the processes in the action phase may also be important for post-action reflection processes.

Finally, our results strengthen the claim of the predictive power of learning approach goals for self-regulated learning processes, congruent with prior research on instructors’ professional learning (Hein et al., 2019; Daumiller et al., 2021c). In our study, learning approach goals predicted later intentions to act on SET-results and intentions to improve teaching that were based on concrete SETs. This study improved the measure of the outcome variable of the post-action phase compared to prior research by including both quantitative and qualitative measures of intentions. The connection between learners’ motivation in the pre-action phase and their intentions formulated in the post-action phase remained robust when controlling for processing time (action phase), low teaching quality, and indicators of the amount of received information. Consequently, the results support the importance of learning goals in the self-regulated learning process of higher education instructors.

### Implications for Educational Practices

Research that sheds light on antecedents of learning from SET-results and the learning process can provide relevant practical implications. As such, fostering learning (approach) goals might be helpful for promoting self-reported learning from higher education instructors’ SETs. Achievement goals of students could be activated by using instructions that emphasize the importance of learning and improvement and by evaluating performance on the basis of changes over time (Elliott and Dweck, 1988; Elliot and Harackiewicz, 1996). This might also be possible for higher education instructors if the quality management includes information that activates learning goals in their communication directly before providing instructors with their SET-results. In addition, the possibility to strengthen learning approach goals in academics by workplace interventions has been discussed in previous literature (Janke and Dickhäuser, 2018). However, as instructors already report high learning approach goals (compared to the midpoint of the scale) and SETs are mostly mandatory in higher education institutions in Germany and further nations (pre-action phase), it might be beneficial to support instructors in the following steps of the learning process (action and post-action phase) to improve professional learning from SETs in educational practice. To support instructors in building intentions in the post-action phase of the learning process, didactical courses might promote *intentions to act* on SETs for further improvement (e.g., by explaining possibilities and advantages to discuss SET-results with colleagues and students, consider changes in future courses, and participate in further relevant didactical trainings). Instead of only informing instructors about the SETs in higher education institutions, *intentions to improve teaching* might be promoted by encouraging instructors to reflect on their SET-results with a short qualitative survey on their goals for future teaching, which they should complete after processing the SETs. To facilitate instructors’ reflections, they could think about different questions concerning their SETs (e.g., what do they learn from the SETs? What do they want to improve in their future teaching and how could they do that?). However, our study does not provide evidence for the causality of the identified associations or the consequences of intentions to act on SET-results and intentions to improve future teaching for later quality of teaching. For these reasons, practical ideas need to be tested in intervention studies before they can be implemented into higher education systems.

### Limitations and Future Directions

Against our hypotheses, we did not find work avoidance goals (or appearance goals in bivariate analyses) to predict taking part in voluntary SET(s). This could, however, be a direct effect of our acquisition strategy that relied on the willingness of instructors to participate in a study where they were meant to interact with SETs. This in itself is a motivated action and instructors with low or suboptimal motivation may have been less likely to participate in the study, limiting our ability to detect effects of this goal type. The observed means for achievement goals speak to this direction: Learning approach goals were descriptively slightly stronger, while appearance avoidance goals were slightly weaker within our sample compared to previous research with less extensive study designs (e.g., compared to a cross-sectional study by Daumiller et al., 2019). Therefore, it could be highly beneficial to investigate the process of learning from SET-results in a less pre-selected sample of instructors in future research through applying more economic study designs. Such a study design may replicate and advance our findings, for example, by questioning university instructors at the beginning of their semester about motivational variables, beliefs, and fears and then measuring relevant outcome variables after they processed their mandatory SETs (rather than additionally asking for them to complete voluntary SETs). Additionally, context characteristics could have impacted the instructors’ decisions to voluntarily conduct SETs (e.g., whether they also had to conduct mandatory SETs in the semester of study participation or not). The evaluation context would also be unified in the above mentioned study, in so far as all instructors would only conduct mandatory evaluations.

Although we tried to prevent biases in processing times by encouraging instructors to look at the results only when they had enough time to process them, by excluding times in which another tab in the browser was viewed, and by excluding participants with unreasonably high processing times, we cannot rule out completely that instructors kept the tab with the SET-results open for other reasons besides looking at them (e.g., leaving the desktop open while getting a coffee). For this reason, the indicator for processing time might be limited in its reliability and validity. This concern in regard to the validity of log data is in line with research on university students which did not find statistically significant associations between self-reported engagement and objective log data in an online learning system (Henrie et al., 2018). Nevertheless, the results of our study at least partially support the validity of this measure, as processing time was associated with possible predictors and outcomes in meaningful ways. In particular, it took the instructors longer to process the results if more students participated and if the teaching quality was rated worse. Moreover, processing time was significantly correlated with the intentions to improve teaching. Future studies that aim to use this objective measure could improve the reliability of processing time further and thereby the estimation of respective associations by letting the instructors process the SET-results under more controlled conditions (e.g., observation).

Furthermore, due to the natural setting of the study, instructors could evaluate their courses in an online tool, and in single courses only one student participated in the students’ evaluation of teaching. Unfortunately, we could not prevent low student participation rates (despite reminders to the instructors to share the invitations with their students and direct reminders to the students when possible). As the validity of SET(s) rises with the participation rate and instructors use this information for the interpretation of SET-results (Nowakowski and Hannover, 2015), the low student participation rate might limit the interpretation of the findings in that the average processing times might underestimate the real amount of time that it takes instructors to process SET-results. To reduce the impact of this variation in our findings, we controlled for the number of students that participated in the course evaluation within the mediation analyses. However, it might be fruitful for future research to take further steps to prevent low response rates in SETs into account (e.g., by additionally asking for in-class evaluations or investigating the processing of obligatory SET-results).

In our study, we mostly focused on the learning process at the beginning rather than on the future learning result. Due to the complex sample (different countries, multiple universities, and different departments), we did not have access to additional objective measures of teaching advancement besides self-reported intentions to improve future teaching. In future research, this limitation could be overcome by focusing on instructors’ concrete goals to improve future teaching based on SETs and assessing their goal attainment in subsequent semesters by self-ratings and external ratings of students or colleagues.

Finally, our results indicate temporal trends, however, they cannot tackle the question of causality, which calls for further experimental studies. In such studies, it would be interesting to investigate how researchers perceive and interact with SETs depending on prior induced achievement goals. In a naturalistic design, the instructors could be briefed to bring their own SETs, while a less extensive solution could be to provide them with vignettes of fictional SETs.

Feedback theories and models of self-regulated learning provide frameworks to look into instructors’ learning from student feedback in future research. Research on student learning provides evidence that the least complex feedback was beneficial for learners in terms of efficiency and learning outcomes (Kulhavy et al., 1985). As the complexity of SET-results is quite high, reducing the complexity of SETs or helping to interpret complex student feedback might be beneficial for instructors’ learning outcomes. This would be of high interest for future research and of practical significance for how to provide SET-results in the evaluation process in higher education institutions. Furthermore, future research could focus on reasons and concrete goals to use SETs to predict the usage of SET(s) and learning from its results.

## Conclusion

The present study provides new insights into higher education instructors’ voluntary usage and learning from student evaluations of teaching. Our results suggest that especially learning goals play an important role in predicting whether instructors voluntarily conduct SETs as well as their intentions to act on the SETs and improve future teaching. Understanding the impact of professional motivation of higher education instructors on the processing and voluntary use of SETs is crucial in fostering instructors’ professional development in teaching. All in all, the ideas presented in this article provide the foundation for future research on instructors’ learning from SET-results with the goal of advising higher education institutions, instructors, and quality management on how to support instructors in seeing SETs as valuable learning opportunities.

## Data Availability Statement

The datasets generated for this study are available on request to the corresponding author.

## Ethics Statement

The studies involving human participants were reviewed and approved by EK Mannheim 28/2018. The patients/participants provided their written informed consent to participate in this study.

## Author Contributions

All authors listed have made a substantial, direct and intellectual contribution to the work, and approved it for publication.

## Conflict of Interest

The authors declare that the research was conducted in the absence of any commercial or financial relationships that could be construed as a potential conflict of interest.

## Footnotes

1 Since SETs contain students’ reports regarding their perception of instructors’ competencies, we assume that especially the appearance component of performance goals is relevant for predicting the use of SETs. Consequently, we focus on the appearance component of performance goals within our study. We did not consider normative goals, as these goals should be relevant when instructors have the clear possibility to compare their results with colleagues (e.g., in a situation with mandatory SETs), which was not the case in our study on voluntary conducted SETs. In addition, possible associations between task goals and professional learning are not clear from a theoretical perspective (Daumiller and Dresel, 2020).

2 This study was part of an overarching three-semester longitudinal study, in which we assessed further constructs. Subsamples of this dataset have already been used to examine research questions concerning the associations of achievement goals and faculty members’ discrete emotions (Rinas et al., 2020), burnout and teaching quality in the shift from face-to-face to online teaching (Daumiller et al., 2021b), self-efficacy and students’ emotions (Daumiller et al., 2021a), and to describe and promote the online tool for evaluations (Janke et al., 2020). The core focus of the present study, namely analyzing the interplay of achievement goals with the instructors’ learning process regarding SETs, has not yet been addressed in any of the previously published manuscripts.

3 https://www.lehr-evaluation-online.de/

4 Higher education institutions in Germany and Austria can be categorized as integrated systems (Kwiek and Antonowicz, 2013) as the academic staff take on tasks in both research and teaching. The academics in both countries spend most hours (on average) on research-related activities (41% of the working hours in Germany and 39% in Austria, Kwiek and Antonowicz, 2013). In both countries, the proportion of temporary positions is high (see Huisman et al., 2002 for details on junior academics’ temporary contracts in Europe), junior academics work under precarious conditions (Gallas, 2018), doctoral candidates conduct teaching (Kwiek and Antonowicz, 2013), and the proportion of doctoral candidates and post-doc positions within the academic staff is high. In addition, the same language is spoken in both countries, and there is significant fluctuation of academic staff between both countries. As the higher education systems and instructors of both countries share common features (see Supplementary Material 1 for results of invariance analyses on the predictor variables in this study), we consider instructors from both countries as equally suitable to be included in our sample.

5 As end of March 2020 constitutes the end of the first semester of study participation for all instructors, we retrieved our data then. Later data cannot be used to test the hypotheses presented within this study, as this data might be biased by the sudden shift to digital teaching as a reaction to the COVID-19 pandemic.

6 We assessed further goals that can be distinguished in instructors according to previous literature (Daumiller et al., 2019). However, as we had no hypotheses for these further differentiated goals, we do not report on them within this paper. Exploratory analyses on associations of further achievement goals and the outcome variables of this study (conducting voluntary SETs, processing time, intentions to act, and intention to improve teaching) are depicted in the Supplementary Material 2. There were no statistically significant associations in latent correlation models.

7 The SET-results consisted of quantitative and qualitative student feedback. The scale scores for the SEEQ scales (e.g., learning/value or group interaction), single items on student background characteristics (e.g., prior subject interest), and open comments by students (e.g., what they liked and what can be improved within the evaluated course) were presented online. Mean scores of the scales of the SEEQ and distribution charts for the student background characteristics were displayed to summarize quantitative feedback, while the single comments of students on open answers were listed under the questions.
