# Supplementary material for: Higher Education Instructors’ Usage of and Learning From Student Evaluations of Teaching – Do Achievement Goals Matter?
Source: Front Psychol. 2021 Jul 20;12:652093. doi: 10.3389/fpsyg.2021.652093 (PMC8329422; doi:10.3389/fpsyg.2021.652093)
Supplement: Supplementary file 1 [file Data_Sheet_1.PDF]

Supplementary Material

1 Measurement Invariance Testing

To confirm that the structure of the baseline predictors (achievement goals, beliefs in the validity of SETs, and general experienced threat through negative feedback) is independent from the country the instructors work in (Germany and Austria), and that instructors from both German-speaking countries share common features, multi-group confirmatory factor analyses (MGCFA) were conducted. Correlated latent factors were estimated for all predictors simultaneously. We tested for configural, metric, and scalar invariance (in a total of three models, adding one additional restriction to each) to ensure an equivalent factor structure, factor loadings, and intercepts for instructors in both countries. When a more restricted model does not describe the data worse than the previous, we assumed the corresponding form of invariance. Cut-off values of at least ΔCFI = .01 paired with changes in RMSEA of .015 and SRMR of .030 (for metric invariance) or .015 (for scalar invariance) have been suggested, because χ^2 is overly sensitive for small deviations in large samples (Chen, 2007; for an overview see Putnick & Bornstein, 2016). The absolute fit indices from the measurement invariance analyses can be found in the Additional Table 1.

Additional Table 1**.** *Results of Measurement Invariance Testing (for all Baseline Predictors).*

	CFI	ΔCFI	TLI	ΔTLI	RMSEA	ΔRMSEA	SRMR	ΔSRMR
Configural invariance	.921	–	.907	–	.064	–	.057	–
Metric invariance	.919	.002	.908	–.001	.063	.001	.066	–.009
Scalar invariance	.919	.000	.910	–.002	.063	.000	.067	–.001

Note. N = 407 (N_{GE} = 262, N_{AU} = 145). Estimator = MLR; Model = configural, metric, scalar. We allowed for eight correlations of error variances for items with similar wordings between learning approach and avoidance goals as well as between appearance approach and avoidance goals.

The results confirmed configural, metric, and scalar invariance for the sub groups of instructors from Germany and Austria. The sub samples of instructors from Germany and Austria share common features regarding their achievement goals, beliefs in the validity of SETs, and general experienced threat through negative feedback. Thereby, the subgroups can be examined as one sample in the further reported analyses.

2 Exploratory Analyses on Further Achievement Goals

Additional Table 2. *Bivariate associations of further achievement goals and outcomes.*

	Conducting SET(s)			Processing time			Intentions to act			Intentions to improve teaching		
Achievement goals	*r*	*SE*	*p*	*r*	*SE*	*p*	*r*	*SE*	*p*	*r*	*SE*	*p*
Task approach	.01	0.07	.866	.14	0.08	.075	.07	0.13	.605	.05	0.10	.581
Task avoidance	.02	0.07	.734	.12	0.07	.074	–.05	0.11	.629	–.12	0.09	.173
Normative approach	–.04	0.07	.580	–.06	0.10	.531	.00	0.11	.999	–.02	0.09	.780
Normative avoidance	.08	0.06	.204	.14	0.09	.118	–.09	0.11	.416	–.01	0.09	.902
Relational	–.08	0.07	.255	.08	0.09	.362	.20	0.11	.078	–.02	0.09	.869
	N = 407			*N* = 152			*N* = 152			*N* = 152		

Notes. *r* = correlation coefficient; *SE* = standard error; *p* = two-tailed level of significance. Significant effects (p <.05) are printed in boldface. The achievement goals and intentions to act were modelled as latent variables. The depicted latent correlations are derived from two models in which undirected paths between all included variables were freed. Model 1 entailed correlations between the achievement goals and conducing SETs (N = 407, estimator = WLSMV, CFI = .90, TLI = .88, RMSEA = .04, SRMR = .04). Model 2 entailed achievement goals and all further outcome variables (N = 152, estimator = MLR, CFI = .93, TLI = .92, RMSEA = .05, SRMR = .06).

3 Group Specific Means

Additional Table 3. *Sub-group Specific Descriptive Statistics (Variables Pre-action Phase).*

		Min	*Max*	*M*	*SD*	N_{Group}
Learning approach goals	– doctoral candidates	1.50	8.00	7.00	1.04	135
	– post-docs	1.00	8.00	7.02	1.05	116
	– professors	1.00	8.00	6.76	1.36	75
	– temporal position	1.00	8.00	7.03	1.12	273
	– permanent position	1.50	8.00	6.82	1.19	112
Learning avoidance goals	– doctoral candidates	1.00	8.00	6.09	1.76	135
	– post-docs	1.00	8.00	6.19	1.77	116
	– professors	1.00	8.00	6.27	1.63	75
	– temporal position	1.00	8.00	6.12	1.82	273
	– permanent position	1.00	8.00	6.28	1.58	112
Appearance approach goals	– doctoral candidates	1.50	8.00	6.17	1.30	135
	– post-docs	1.00	8.00	6.10	1.42	116
	– professors	1.75	8.00	5.71	1.69	75
	– temporal position	1.00	8.00	6.12	1.44	273
	– permanent position	1.75	8.00	5.89	1.51	112
Appearance avoidance goals	– doctoral candidates	1.00	8.00	6.17	1.90	135
	– post-docs	1.00	8.00	6.08	1.76	116
	– professors	1.00	8.00	5.82	2.05	75
	– temporal position	1.00	8.00	6.07	1.93	273
	– permanent position	1.00	8.00	6.01	1.97	112
Work avoidance goals	– doctoral candidates	1.00	8.00	3.13	1.91	135
	– post-docs	1.00	7.25	2.40	1.47	116
	– professors	1.00	8.00	2.89	1.76	75
	– temporal position	1.00	8.00	2.84	1.76	273
	– permanent position	1.00	7.25	2.59	1.70	112
Validity Beliefs	– doctoral candidates	1.80	5.00	3.61	0.70	135
	– post-docs	1.40	5.00	3.48	0.68	116
	– professors	1.00	5.00	3.53	0.80	75
	– temporal position	1.00	5.00	3.55	0.72	273
	– permanent position	1.40	5.00	3.54	0.79	112
Experienced Threat	– doctoral candidates	1.25	6.00	3.01	1.12	135
	– post-docs	1.00	5.75	3.03	1.01	116
	– professors	1.00	5.75	2.86	0.99	75
	– temporal position	1.00	6.00	3.07	1.11	273
	– permanent position	1.00	4.50	2.62	0.86	112

Notes. Min = Minimal; *Max* = Maximum; *M* = Mean; *SD* = Standard deviation for the sub groups in regard to academic status and employment situation. $N_{\text{ACADEMIC STATUS}}$ = 326 (81 missings); $N_{\text{EMPLOYMENT SITUATION}}$ = 385 (22 missings).

Additional Table 4. *Sub-group Specific Descriptive Statistics (Variables Post/Action Phases).*

		Min	*Max*	*M*	*SD*	N_{Group}
Processing time in minutes	– doctoral candidates	0.54	31.17	5.10	4.72	47
	– post-docs	0.15	27.29	4.23	5.17	38
	– professors	1.17	7.78	3.50	1.72	16
	– temporal position	0.15	31.17	4.68	4.89	88
	– permanent position	1.17	7.38	3.40	1.47	31
Intentions to act	– doctoral candidates	1.33	4.67	3.10	0.86	48
	– post-docs	1.17	4.50	2.90	0.80	41
	– professors	1.67	3.83	2.72	0.65	19
	– temporal position	1.17	4.67	2.98	0.83	92
	– permanent position	1.50	4.33	2.89	0.66	34
Intentions to improve teaching	– doctoral candidates	0.00	5.00	1.48	1.19	48
	– post-docs	0.00	4.50	1.34	1.11	41
	– professors	0.00	4.50	0.97	1.05	19
	– temporal position	0.00	5.00	1.41	1.13	92
	– permanent position	0.00	4.50	1.18	0.98	34
Low teaching quality	– doctoral candidates	1.00	3.25	1.98	0.58	52
	– post-docs	1.00	2.92	1.77	0.46	43
	– professors	1.00	3.00	1.91	0.53	25
	– temporal position	1.00	4.50	1.88	0.60	97
	– permanent position	1.00	3.00	1.85	0.48	44
Number of courses	– doctoral candidates	1.00	3.00	1.04	0.27	53
	– post-docs	1.00	2.00	1.09	0.29	44
	– professors	1.00	4.00	1.36	0.81	25
	– temporal position	1.00	3.00	1.08	0.34	99
	– permanent position	1.00	4.00	1.23	0.60	44
Number of students	– doctoral candidates	1.00	45.00	10.65	7.97	53
	– post-docs	1.00	42.00	9.75	8.26	44
	– professors	1.00	45.00	11.44	10.53	25
	– temporal position	1.00	45.00	9.75	8.09	99
	– permanent position	1.00	45.00	11.39	9.17	44
Additional questions	– doctoral candidates	0.00	15.00	0.98	2.51	53
	– post-docs	0.00	12.00	0.90	2.16	44
	– professors	0.00	3.00	0.54	0.94	25
	– temporal position	0.00	15.00	1.12	2.59	99
	– permanent position	0.00	5.00	0.53	1.18	44

Notes. Min = Minimal; *Max* = Maximum; *M* = Mean; *SD* = Standard deviation, for the sub groups in regard to academic status and employment situation. $N_{\text{ACADEMIC STATUS}}$ = 122 (30 missings); $N_{\text{EMPLOYMENT SITUATION}}$ = 143 (9 missings).
